# The Instructive Role of the Bone Marrow Niche in Aging and Leukemia

**DOI:** 10.1007/s40778-018-0143-7

**Published:** 2018-10-12

**Authors:** Elisa Lazzari, Jason M. Butler

**Affiliations:** 1 Center for Discovery and Innovation, Hackensack University Medical Center, Nutley, NJ 07110 USA; 2 Lombardi Comprehensive Cancer Center, Georgetown University, Washington, DC 20057 USA

**Keywords:** BM niche, Endothelial cells, Hematopoiesis, Aging, Myeloid leukemia

## Abstract

**Purpose of review:**

In this review, we aim to discuss the role of the bone marrow microenvironment in supporting hematopoiesis, with particular focus on the contribution of the endothelial niche in dictating hematopoietic stem cell (HSC) fate.

**Recent findings:**

Evidence gathered in the past two decades revealed that specific cell types within the bone marrow niche influence the hematopoietic system. Endothelial cells have emerged as a key component of the HSC niche, directly affecting stem cell quiescence, self-renewal, and lineage differentiation. Physiological alterations of the bone marrow niche occurring in aging have been described to be sufficient to promote functional aging of young HSCs. Furthermore, a growing body of evidence suggests that aberrant activation of endothelial-derived signaling pathways can aid or trigger neoplastic transformation.

**Summary:**

Several groups have contributed to the characterization of the different cell types that comprise the complex bone marrow environment, whose function was long perceived as an undiscernible sum of many parts. Further studies will need to uncover niche cell-type-specific pathways, in order to provide new targets and therapeutic options that aim at withdrawing the microenvironmental support to malignant cells while sparing normal HSCs.

## Introduction

For many years, the bone marrow (BM) niche was considered an inert scaffold for hematopoietic stem cells (HSCs), but in the past two decades, it has been recognized as a complex and dynamic tissue providing the right “soil” to ensure the fulfillment of the HSC potential. Many molecular and cellular components contribute to this plastic environment. In this review, we discuss the recent advances that had shed light on the complexity of the BM niche; in particular, we will focus on the role of the endothelial niche, which sustain HSCs throughout their lifespan, since the emergence of definitive HSCs during embryonic development. Furthermore, we aim at reviewing how the process of aging re-shapes the BM endothelial niche and consequently affects hematopoiesis, impairing tissue homeostasis. Advancing age is irrefutably accompanied by an increased incidence of cancer, thus making critical the discussion on how alterations of the BM microenvironment may instruct and/or support the emergence of aberrant disease-initiating leukemia stem cells (LSC). Here, we will examine several lines of evidence that associate the genetic changes in BM non-hematopoietic cells and the leukemia-induced BM niche remodeling with the emergence of leukemia cells endowed with stem cell-like properties.

## The Hematopoietic Stem Cell Niche

In the mouse embryo, definitive HSCs emerge in the aorta-gonad-mesonephros (AGM) region around embryonic day (E) 11 [1], as a result of endothelial-to-hematopoietic transitions [2–4]. These HSCs then migrate to the fetal liver, where they undergo a considerable expansion [5]. Eventually, HSCs colonize the BM concurrently with marrow vascularization (E16.5) [6]. The BM niche thereafter provides a critical multicellular microenvironment throughout adult life, regulating HSC quiescence, proliferation, mobilization, and lineage differentiation. In addition to endothelial cells, other cell types are determinant for the life-long regeneration of the blood system, including perivascular mesenchymal stem cells [7–9], adipocytes [10], mature osteoblasts [11•, 12•], non-myelinating Schwann cells [13], sympathetic nerves [14, 15], and hematopoietic-derived differentiated cells such as megakaryocytes [16, 17], granulocytes [18], and regulatory T cells [19, 20].

It was initially presumed that the HSC niche comprised mainly osteolineage cells [21, 22], whereas the BM vasculature served as a critical cellular hub for the regeneration of the hematopoietic system following myelosuppressive damage [23]. However, technical advances in imaging and the refinement of cell surface markers enriching for HSCs demonstrated that quiescent HSCs reside in close proximity to vascular niches [24, 25], including endothelial cells and perivascular cells [26]. Furthermore, the conditional deletion of key factors, such as stem cell-active cytokine (C-X-C motif) ligand 12 (CXCL12, also known as SDF1α) from mineralized osteoblasts or *Osterix*-expressing osteoprogenitor cells [11•], and of stem cell factor (SCF, known as KITL) from *Col2.3*-expressing osteoblasts [12•] has been reported to not significantly affect HSC frequency and function. Notably, these findings showed that the expression of CXCL12 from cells in the perivascular region, including endothelial cells and mesenchymal progenitors [11•], and of SCF from leptin receptor (Lepr)-expressing perivascular stromal cells and endothelial cells [12•] supported HSCs. The hypothesized contribution of mature osteolineage cells to the maintenance of HSC was additionally lessened by more recent studies combining whole-mount confocal immunofluorescence imaging with 3D computational modeling. These studies found that HSCs preferentially localized in endosteal zones, in close contact with microvessels [27, 28••], consistent with an ancillary role for mature osteoblasts in HSC control. Rather, it emerged that immature osteoprogenitor cells are HSC regulators. These osteolineage-committed progenitors are comprised in mesenchymal stem cells and are often localized in perivascular regions [29].

## The Bone Marrow Endothelial Niche

Endothelial cells are specialized cells that form the inner lining of all blood vessels and support tissue growth and repair [30]. Within the BM microenvironment, endothelial cells are organized in a hierarchical structure of central, longitudinal arteries that give rise to smaller arterioles, which transition to the venous circulation in proximity of the endosteum, and then into sinusoid vessels that extend back in the BM cavity [27]. Recent data has postulated that the endothelial niche can be divided into two distinct pro-HSC niches, the arteriolar niche that is identified by the VEcadherin^+^ (CDH5) CD31^+^ Endomucin^+/−^ SCA1^high^ (Ly6a) VEGRF3^−^ (FLT4) surface phenotype and the sinusoidal niche, identified by VEcadherin^+^ CD31^+^ Endomucin^+^ SCA1^low^ VEGRF3^+^ [28••, 31, 32]. In the context of SCF, it has been suggested the arteriole cells are responsible for the maintenance of the HSC [33]; however, it is still unclear whether these two subcategories play different instructive roles in HSC function in regard to other known or as of yet discovered pro-HSC factors [8, 17, 28••, 33–35].

The role of the BM endothelium in regulating the hematopoietic system first emerged in the context of hematopoietic recovery after myelosuppression [31, 35]. Studies in mice revealed that 5-fluorouracil (5-FU) treatment led to an increase in soluble KITL and plasma vascular endothelial growth factor A (VEGF-A) levels along with the expansion of *Tie2*-positive neovessels in the adult BM. Moreover, inhibition of Tie2 signaling contributed to impaired neoangiogenesis, leading to a delay in hematopoietic recovery [35]. The critical importance of vessel maintenance to functionally support HSCs was further demonstrated by the conditional deletion of *Vegfr2* in adult mice, which was shown to block regeneration of sinusoidal ECs in sublethally irradiated animals, thus preventing hematopoietic reconstitution [31]. Furthermore, inhibition of VEGFR2 using antibodies prevented regeneration of damaged sinusoidal ECs, thereby interfering with the engraftment of HSCs and leading to hematopoietic failure in lethally irradiated mice [31].

Pioneering work from our group demonstrated that endothelial cells have the capacity to release angiocrine factors to support the in vitro self-renewal and in vivo reconstitution of long-term (LT) HSC pool after myeloablation [36••]. Specifically, activation of Akt-mTOR pathways in endothelial cells upregulated stem cell active angiocrine factors that supported the expansion of HSCs. Conversely, MAPK signaling in endothelial cells shifted the balance towards HSC differentiation, thus suggesting that the endothelial niche could support both self-renewal and lineage-specific differentiation of HSCs [37]. The expression of Notch ligands Jagged-1 and Jagged-2 by BM-derived endothelial cells (BMECs) also regulates HSC proliferation and quiescence [37, 38•, 39]. Endothelial-specific deletion of Jagged-1 using *VE-cadherin-Cre* mice resulted in a significant decrease in the number of phenotypic LT-HSCs at steady state, and in inhibited hematopoietic regeneration after sublethal irradiation [38•]. Notably, Jagged-2 expression in BMECs was dispensable for maintaining the capacity of hematopoietic stem and progenitor cells (HSPCs) to repopulate under steady-state conditions, but contributed to their recovery in response to myelosuppressive injury, by activating Notch 2 [39]. Recently, the activation of Notch pathway has also been described as a positive regulator of vascular growth in adult bone. In particular, a new capillary subtype, defined as a rare CD31^high^Endomucin^high^ or “Type H” endothelium has been implicated in osteogenesis and in chondrocyte maturation [32, 40]. This endothelial cell subset was enriched in the bone metaphysis and at the endosteal surface and marked the distal end of CD31^+^Endomucin^−^ arterioles. However, an independent multivariate flow cytometry analysis of adult BM utilizing intravital injection of VE-cadherin and endothelial reporter mice demonstrated that both arterioles and sinusoids were CD31^high^, and that all VEGFR3^+^ sinusoids and a subset of arterioles were Endomucin^high^ [41]. Similarly, a novel endoglin (CD105)-expressing endothelial cell subpopulation was described in human BM upon regeneration after chemotherapeutic injury. These CD105-expressing endothelial cells were a rare fraction of CD31^+^ endothelial cells in steady-state adult BM but increased significantly (13–19-fold change increase) upon administration of 5-FU. These findings confirmed a relative increase of this subset in the regenerative phase after myeloablation, thus leading to designate these endothelial cells “human regeneration-associated ECs” (hRECs). hRECs harbor several similarities to the recently identified Type H endothelium in mice, including localization to the bone surface and reduced frequencies during aging [32, 40, 42].

Overall, these findings are particularly noteworthy in relation to the myelosuppressive preparative regimens for hematopoietic stem cell transplantation (HSCT). Indeed, pre-conditioning regimens for blood malignancies and autoimmune disorders can cause systemic endothelial damage and have been associated with HSCT-related complications, including acute graft-versus-host disease, through endothelial apoptosis and pro-inflammatory cytokine production [43, 44, 45•]. Chronic inflammatory signaling is a renewed factor implicated in impaired HSC repopulation potential [46–48]. Therefore, strategies aimed at limiting pro-inflammatory responses that affect HSC maintenance and BM endothelial niche integrity may be an attractive option to preserve long-term HSC function following hematopoietic injury. To this aim, we have recently identified the canonical NF-kB pathway as an extrinsic mediator of HSC function within the adult BM endothelial niche. Endothelial-specific inhibition of canonical NF-kB signaling using a dominant negative IkB-SS construct expressed under *Tie2* promoter elements resulted in a profound increase in HSC self-renewal and regenerative potential [49]. More notably, the infusion of exogenous [50••, 51] NF-kB-inhibited BMECs in irradiated mice resulted in a significant radio-protective effect that mitigated hematopoietic damage [49]. Furthermore, we have demonstrated that co-infusion of young BMECs with whole BM transplants can shepherd the transplant to the BM microenvironment resulting in an increase in the engraftment potential of a limited number of donor hematopoietic cells, as well as restore the functionality of transplanted aged donor cells [52••]. Taken together, these findings underline the considerable potential of therapeutic approaches targeting the endothelial niche, either by the direct modulation of niche-specific signaling or by infusion of ex vivo expanded cellular therapeutics.

## Aging of the Bone Marrow Niche and its Effects on Hematopoiesis

It is generally believed that aging results in the progressive decrease of stem cell reservoirs; although, when looking at the hematopoietic system, aging entails complex and somehow counterintuitive alterations. While the absolute number of phenotypically defined HSCs increases with age, aged HSCs exhibit decreased self-renewal and reconstitution capacity [53, 54, 55•, 56–59]. In particular, aged LT-HSC show a myeloid-biased differentiation potential compared with young HSC, and diminished lymphoid potential [54, 55•, 56, 57].

Aged HSCs express elevated levels of genes associated with nitric oxide (NO)-mediated signal transduction, stress response, inflammation [53], as well as with myeloid lineage differentiation, cell cycle [60, 61] and proliferation (including TGFβ and ERK/MAPK pathways) [56, 60]. In contrast, downregulated genes often include those involved in the preservation of genomic integrity, such as chromatin remodeling, DNA repair [53] and DNA replication. Cycling aged HSCs in mice have shown elevated levels of replication stress associated with cell cycle defects [62], reduced cell polarity, and adhesion to the BM niche [63, 64]. In all, most observations in regard to the aging of the hematopoietic system have focused on cell intrinsic changes in HSCs which can portend to leukemic transformation, but whether BM niche age-associated alterations further exacerbate or even are sufficient to initiate blood disorders is still unclear.

**Fig. 1:**
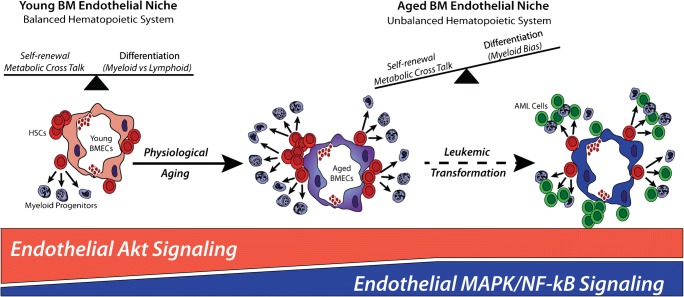
Representative diagram of the instructive role of the BM vascular niche towards HSC regulation, demonstrating that the activation of Akt signaling in young ECs aids HSC function. As aging occurs, the activation state of EC shifts towards pro-inflammatory pathways, including MAPK and NF-kB signaling, leading to impaired HSC function, while promoting leukemia cell expansion

Recent evidence has demonstrated that cell extrinsic alteration can support the onset of HSC aging phenotypes. Indeed, it has been shown that the myeloid lineage skewing of aged HSCs is associated to increased secretion of the pro-inflammatory CC-chemokine ligand 5 (CCL5, also known as RANTES) by aged stroma [65]. Similarly, reduced expression of secreted matrix protein osteopontin (OPN) in aged stroma confers aging-associated phenotypes to HSCs, including loss of cell polarity and impaired engraftment potential [66]. Additionally, it has been demonstrated that loss of BM innervation by the sympathetic nervous system can promote premature aging of young HSCs [67]. In line with these findings, our group has recently demonstrated that in both in vitro and in vivo settings, aged BMECs can instruct an aged phenotype in young HSCs, whereas young BMECs can partially rejuvenate aged HSC function [52••]. These data suggest that aged BMECs could be primed to negatively affect HSC function, but that exposure to angiocrine factors from young BMECs can aid in improving aged HSC function. Indeed, it has been demonstrated that aged BMECs have compromised function in regulating hematopoietic niche cells and that it is possible to rejuvenate their functional readout by mitigating the activation of endothelial Notch signaling; however, these studies were unable to restore the full functional capacity of HSCs [42]. These findings strongly suggest that deleterious changes in aged BM microenvironment inefficiently sustain HSC homeostasis which may lead to aged-related hematopoietic disorders (Fig. 1), providing intriguing models to speculate that a “pre-malignant” niche might induce aged HSCs to undergo malignant transformation into tumor cells.

## The Leukemic Bone Marrow Niche

As previously discussed**,** a growing body of evidence demonstrates the critical interactions between healthy HSCs and their niche. Several groups have explored the concept of niche-initiated disease in animal models, showing that alterations of specific BM microenvironment components are sufficient to initiate or set the stage for leukemia. For the scope of this review, we will discuss in more detail experimental models of age-related myeloid disorders, including myeloproliferative neoplasms (MPNs), myelodysplastic syndrome (MDS), and acute myelogenous leukemia (AML). While several studies provided remarkable proof-of-concept in mice, it is still unclear whether a single alteration within the BM microenvironment is sufficient to trigger leukemia in humans, although few groups have recently made indirect observations that support this hypothesis. One of the first striking model of niche-initiated disease was the mesenchymal osteoprogenitor-specific deletion of the miRNA processing endonuclease Dicer1 under the *Osterix* promoter, which induced impaired osteoblast differentiation [68]. Abnormal osteoprogenitor cells were thus implicated in defective HSC function and in key features of human MDS, including peripheral cytopenia, dysgranulopoiesis, dysplastic megakaryocytes, and the emergence of AML [68]. Interestingly, the osteoprogenitor-specific deletion of *Sbds*, the gene mutated in Shwachman-Bodian-Diamond Syndrome, a condition characterized by BM failure and leukemia predisposition, largely phenocopied *Dicer1* deletion [68]. Consistent with this experimental model, decreased expression of DICER1 was detected in mesenchymal stromal cell-derived osteoprogenitors from MDS patients compared to healthy individuals, along with a reduction of SBDS [69].

The altered differentiation of myeloid and lymphoid progenitors was described in mice with osteoblast-specific constitutive activation of β-catenin, leading to the development of AML [70]. Notably, β-catenin stimulated the expression of Jagged-1 in osteoblasts, triggering aberrant Notch signaling in HSC progenitors [70]. These observations were relevant to human disease, as nuclear localization of β-catenin in osteoblasts was found in a cohort of patients with AML or MDS, associated with concurrent Notch signaling activation in hematopoietic cells [70].

Mutations of the protein tyrosine phosphatase SHP2 (encoded by *PTPN11*), a positive regulator of the RAS pathway, in mesenchymal stem/progenitor cells and osteoprogenitors, but not in differentiated osteoblasts or endothelial cells induced MPN [71]. Conversely, the loss of signal-induced proliferation-associated gene 1 (*Sipa1*), a RAP1 GTPase-activating protein expressed mainly by mesenchymal stem/progenitor cells and endothelial cells induced significant alterations in the BM niche prior to the initiation of MDS/MPN in mice [72]. Importantly, transplantation of normal *Sipa1*^+/+^ hematopoietic cells in *Sipa1*^−/−^ recipients was followed by neoplastic transformation in MDS/MPN, recapitulating the same disease features observed in aged *Sipa1*^−/−^ mice, including anemia, increased granulocytes, pronounced B-lymphopenia, and splenomegaly [72]. *Sipa1*-deficient niche cells displayed enrichment of G protein signal pathways, including Ras and Rap1, and of pro-inflammatory cytokines such as transforming growth factor-β (TGFβ) and tumor necrosis factor-α (TNFα) [72]. Overall, the global dysregulation of inflammatory cytokines in the aged/pre-tumoral niche appears to be a critical precondition in the pathogenesis of myeloid disorders.

In another model, loss of canonical Notch signaling in BM stromal and endothelial cells induced significant alterations of hematopoiesis. Specifically, the conditional inhibition of the Notch pathway transcriptional repressor recombination signal-binding protein for immunoglobulin kappa J region (RBPJk) in *Mx1*-expressing cells induced a MPN-like disease, driven by the constitutive upregulation of miR-155 and its downstream activation of NF-kB signaling. *Mx1-RBPJ*^−/−^ mice displayed a significant increase in the levels of inflammatory cytokines G-CSF and TNFα in BM MSCs and ECs [73••]. Notably, endothelial-specific loss of Notch signaling in *Tie2*-*RBPJ*^*−/−*^ mice led to myeloid cell expansion and inflammatory cytokines similar to those observed in *Mx1-RBPJ*^−/−^ mice, despite the moderate reduction of RBPJ in endothelial cells (30%) [73••]. Circulating endothelial progenitor cells isolated from patients with MPN were reported to display the same mutation signature detectable in the malignant hematopoietic clone, including the activating mutation of the Janus kinase 2, JAK2^V617F^ [74]. These findings are particularly interesting in the light of recent observations made in *Tie2*-JAK2^V617F^ mice, where JAK2^V617F^ was specifically expressed in hematopoietic cells and endothelial cells [75, 76•]. JAK2^V617F^-expressing vascular niche promoted the expansion of JAK2^V617F^ leukemia-initiating cells over JAK2^WT^ HSCs in competitive BM transplants, and upregulated the expression of angiocrine factors CXCL12 and SCF compared to wild-type ECs [76•]. Taken together, the indirect observations in human leukemia seem to corroborate the hypothesis that alterations within the non-hematopoietic compartment are sufficient to aid the emergence of LSC. A question of great interest is whether BM niche cells harbor the same “first hit” mutations as pre-leukemic HSCs do, a claim particularly intriguing in the case of the vascular niche, in light of the close ancestral relationship between ECs and HSCs during development. Very fascinating aspects of the LSC-niche relationship have latterly emerged, including the composition and biophysical properties of the extracellular matrix (ECM), and the leukemic cell response to hypoxia within the BM microenvironment. CD98, an ECM protein mediating several integrin-associated adhesive signals, was recently showed to enable the propagation of AML cells [77]. Loss of CD98 after tamoxifen treatment of *Cd98hc*^*fl/fl*^*;Rosa26-CreER* mice prevented leukemia cells to form stable interactions with blood vessels in vivo, as well as in in vitro co-cultures of cKit^+^ leukemia cells with human umbilical vein ECs (HUVECs). Moreover, antibody-mediated blocking of integrin ligand VCAM-1 in endothelial cells had no effect on the adhesion of CD98-deficient cKit^+^ leukemia cells, while it significantly reduced the attachment of wild-type cKit^+^ cells, suggesting that CD98 is required for VCAM-1/integrinVLA-4-mediated interactions between leukemia cells and blood vessels [77]. These results are consistent with previous work from our group, demonstrating that leukemic cells cultured in direct cellular contact with primary human ECs [78] were enriched in LSCs, and exhibited a more aggressive AML phenotype when transplanted in mice [79•]. Stimulation of primary ECs with VEGF-A led to increased LSCs expansion, augmented viability after chemotherapy treatment with Ara-C, and increased adhesion to the endothelium, partially mediated by VLA-4 [79•]. Other groups recently substantiated the role of the vascular niche in human AML patient-derived xenografts (PDX), using unconditioned recipient mice to prevent irradiation-induced toxicity in the BM vasculature. AML PDX displayed an increased number of ECs associated with arterioles (CD31^+^ Sca1^high^), a loss of ECs associated with sinusoids (CD31^+^Sca1^low^), and increased leakiness in the BM [80••]. Gene set enrichment analysis (GSEA) of ECs upon AML engraftment underlined several altered pathways, including angiogenesis, and response to hypoxia. Interestingly, *Nox4*, a NADPH oxidase involved in the response to hypoxia via production of reactive oxygen species (ROS) and release of NO, was upregulated in ECs, consistent with increased levels of NO in the BM of AML xenografts compared with non-transplanted mice or mice engrafted with normal HSCs [80••].

## Conclusions

Understanding the instructive role of the BM niche in normal hematopoiesis is a stepping stone for the development of new therapies in aging and blood malignancies. Future studies will benefit from taking advantage of novel in vivo imaging and functional assays, as well as from a more systematic analysis of non-hematopoietic cells from diagnostic biopsies. Mouse models in the last decade have suggested that a niche cell-associated sensitivity in the emergence of different neoplasms may exist and revealed how LSCs hijack the HSC marrow environment to gain survival benefits. BM inflammation and alteration of vasculature permeability have emerged as key factor in the initiation and development of hematological malignancies. Future work is required to further address the cell type-specific functions and the associated signaling pathways and reciprocal metabolic interactions among the niche components. Advances in this regard will provide us with putative targets to reverse age-dependent alterations of HSC number and function and to predict critical factors involved leukemogenesis.
